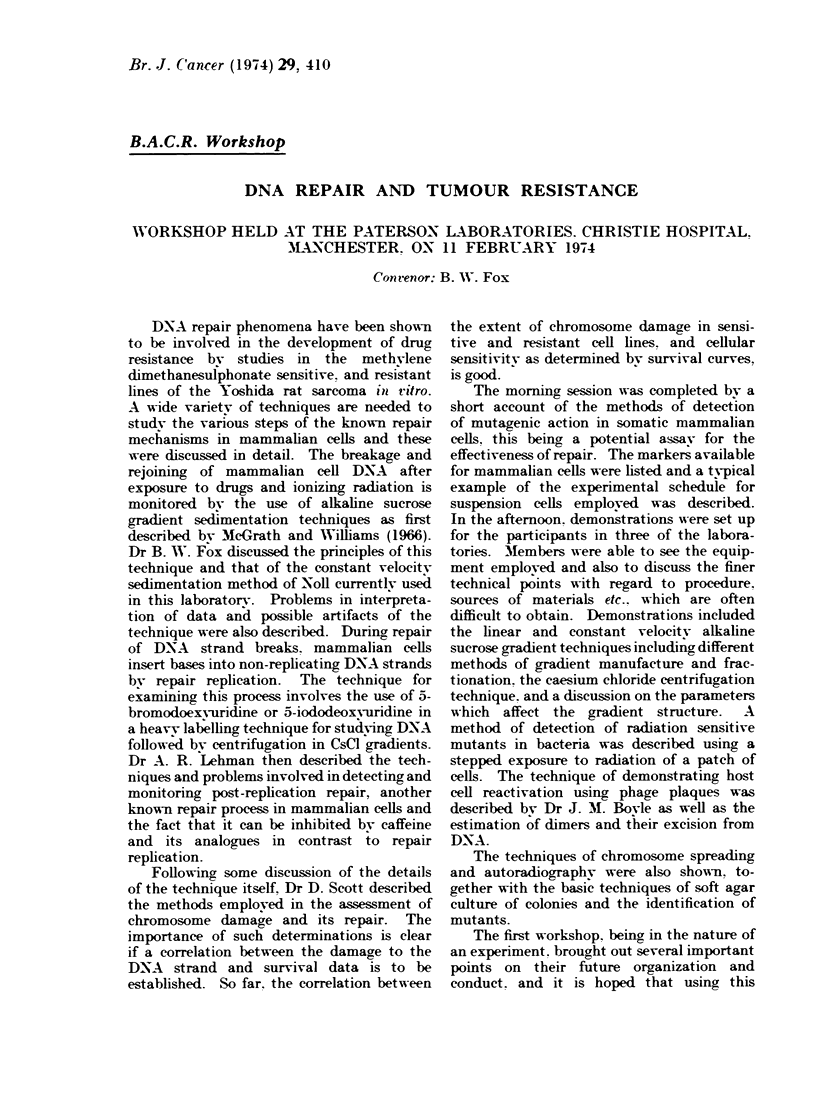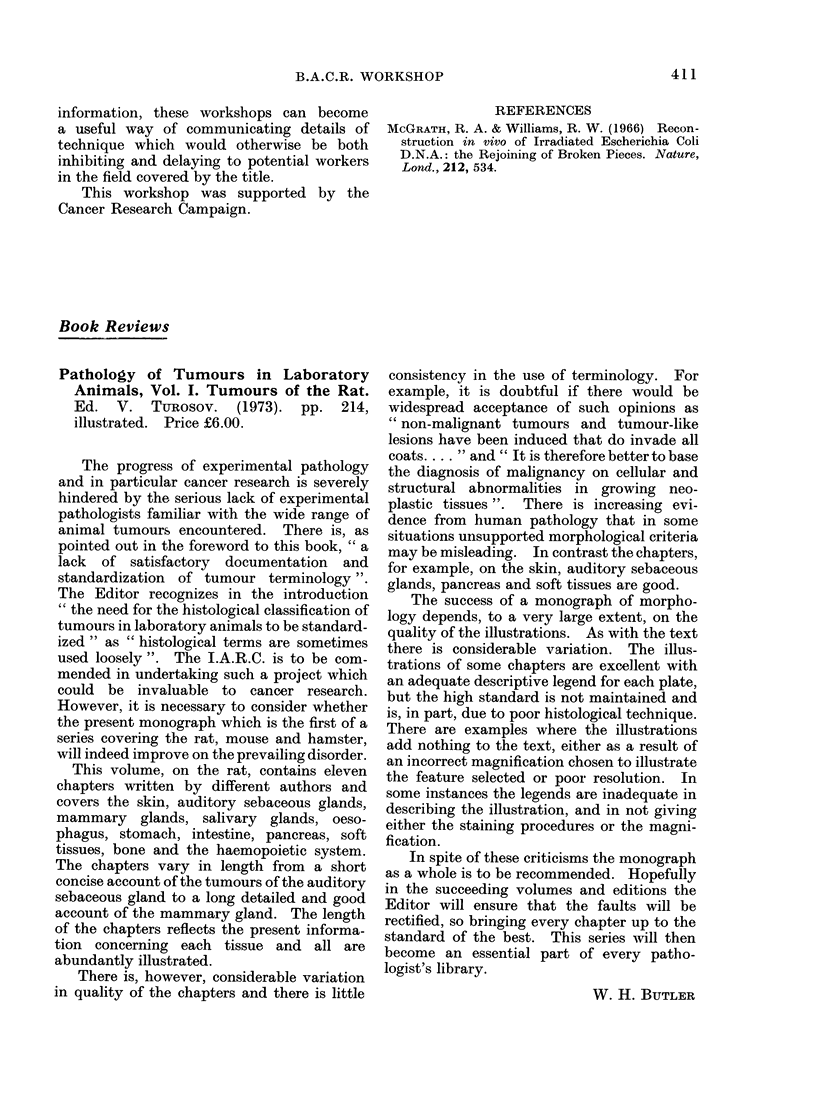# DNA Repair and Tumour Resistance

**Published:** 1974-05

**Authors:** 


*Br. J. Cancer* (1974) **29**, 410

### *B.A.C.R. Workshop*

### DNA REPAIR AND TUMOUR RESISTANCE

### WORKSHOP HELD AT THE PATERSON LABORATORIES, CHRISTIE HOSPITAL, MANCHESTER, ON 11 FEBRUARY 1974

*Convenor:* B. W. Fox

DNA repair phenomena have been shown to be involved in the development of drug resistance by studies in the methylene dimethanesulphonate sensitive, and resistant lines of the Yoshida rat sarcoma *in vitro*. A wide variety of techniques are needed to study the various steps of the known repair mechanisms in mammalian cells and these were discussed in detail. The breakage and rejoining of mammalian cell DNA after exposure to drugs and ionizing radiation is monitored by the use of alkaline sucrose gradient sedimentation techniques as first described by McGrath and Williams (1966). Dr B. W. Fox discussed the principles of this technique and that of the constant velocity sedimentation method of Noll currently used in this laboratory. Problems in interpretation of data and possible artifacts of the technique were also described. During repair of DNA strand breaks, mammalian cells insert bases into non-replicating DNA strands by repair replication. The technique for examining this process involves the use of 5-bromodoexyuridine or 5-iododeoxyuridine in a heavy labelling technique for studying DNA followed by centrifugation in CsCl gradients. Dr A. R. Lehman then described the techniques and problems involved in detecting and monitoring post-replication repair, another known repair process in mammalian cells and the fact that it can be inhibited by caffeine and its analogues in contrast to repair replication.

Following some discussion of the details of the technique itself, Dr D. Scott described the methods employed in the assessment of chromosome damage and its repair. The importance of such determinations is clear if a correlation between the damage to the DNA strand and survival data is to be established. So far, the correlation between the extent of chromosome damage in sensitive and resistant cell lines, and cellular sensitivity as determined by survival curves, is good.

The morning session was completed by a short account of the methods of detection of mutagenic action in somatic mammalian cells, this being a potential assay for the effectiveness of repair. The markers available for mammalian cells were listed and a typical example of the experimental schedule for suspension cells employed was described. In the afternoon, demonstrations were set up for the participants in three of the laboratories. Members were able to see the equipment employed and also to discuss the finer technical points with regard to procedure, sources of materials *etc.*, which are often difficult to obtain. Demonstrations included the linear and constant velocity alkaline sucrose gradient techniques including different methods of gradient manufacture and fractionation, the caesium chloride centrifugation technique, and a discussion on the parameters which affect the gradient structure. A method of detection of radiation sensitive mutants in bacteria was described using a stepped exposure to radiation of a patch of cells. The technique of demonstrating host cell reactivation using phage plaques was described by Dr J. M. Boyle as well as the estimation of dimers and their excision from DNA.

The techniques of chromosome spreading and autoradiography were also shown, together with the basic techniques of soft agar culture of colonies and the identification of mutants.

The first workshop, being in the nature of an experiment, brought out several important points on their future organization and conduct, and it is hoped that using this information, these workshops can become a useful way of communicating details of technique which would otherwise be both inhibiting and delaying to potential workers in the field covered by the title.

This workshop was supported by the Cancer Research Campaign.

REFERENCES

McGrath, R. A. & Williams, R. W. (1966) Reconstruction *in vivo* of Irradiated Escherichia Coli D.N.A.: the Rejoining of Broken Pieces. *Nature, Lond.*, **212**, 534.